# Fixed-target pump–probe SFX: eliminating the scourge of light contamination

**DOI:** 10.1107/S2052252524005591

**Published:** 2024-07-09

**Authors:** Guillaume Gotthard, Andrea Flores-Ibarra, Melissa Carrillo, Michal W. Kepa, Thomas J. Mason, Dennis P. Stegmann, Bence Olasz, Magdalena Pachota, Florian Dworkowski, Dmitry Ozerov, Bill F. Pedrini, Celestino Padeste, John H. Beale, Przemyslaw Nogly

**Affiliations:** a Institute of Molecular Biology and Biophysics ETH Zurich Rämistrasse 101 8092Zürich Switzerland; b Laboratory of Biomolecular Research Paul Scherrer Institut Forschungsstrasse 111 5232Villigen Switzerland; c Swiss Light Source Paul Scherrer Institut Forschungsstrasse 111 5232Villigen Switzerland; d Dioscuri Center for Structural Dynamics of Receptors, Faculty of Biochemistry, Biophysics and Biotechnology Jagiellonian University Gronostajowa 7 30-380Krakow Poland; e Laboratory of Nanoscale Biology Paul Scherrer Institut Forschungsstrasse 111 5232Villigen Switzerland; f Laboratory for Synchrotron Radiation and Femtochemistry Paul Scherrer Institut Forschungsstrasse 111 5232Villigen Switzerland; g Science IT Infrastructure and Services Paul Scherrer Institut Forschungsstrasse 111 5232Villigen Switzerland; h Laboratory for X-ray Nanoscience and Technologies Paul Scherrer Institut Forschungsstrasse 111 5232Villigen Switzerland; Uppsala University, Sweden

**Keywords:** time-resolved crystallography, fixed targets, X-ray free-electron lasers, room-temperature crystallography, pump–probe, photoreceptor light–oxygen–voltage domains, serial femtosecond crystallography, light contamination, sample consumption

## Abstract

X-ray free-electron laser (XFEL) light sources have enabled the rapid growth of time-resolved structural experiments, which provide crucial information on the function of macromolecules and their mechanisms. Here, the aim was to commission the SwissMX fixed-target sample-delivery system at the SwissFEL Cristallina experimental station using the PSI-developed micro-structured polymer (MISP) chip for pump–probe time-resolved experiments. To characterize the system, crystals of the light-sensitive protein light–oxygen–voltage domain 1 (LOV1) from *Chlamydomonas reinhardtii* were used. Using different experimental settings, the accidental illumination, referred to as light contamination, of crystals mounted in wells adjacent to those illuminated by the pump laser was examined. It was crucial to control the light scattering from and through the solid supports otherwise significant contamination occurred. However, the results here show that the opaque MISP chips are suitable for defined pump–probe studies of a light-sensitive protein. The experiment also probed the sub-millisecond structural dynamics of LOV1 and indicated that at Δ*t* = 10 µs a covalent thio­ether bond is established between reactive Cys57 and its flavin mononucleotide cofactor. This experiment validates the crystals to be suitable for in-depth follow-up studies of this still poorly understood signal-transduction mechanism. Importantly, the fixed-target delivery system also permitted a tenfold reduction in protein sample consumption compared with the more common high-viscosity extrusion-based delivery system. This development creates the prospect of an increase in XFEL project throughput for the field.

## Abstract 2

Time-resolved serial femtosecond crystallography experiments can be performed with samples delivered on solid supports. Sample consumption is significantly reduced when compared with the popular crystal-delivery system via high-viscosity extrusion.

## 1. Introduction

The emergence of serial femtosecond crystallography (SFX) at X-ray free-electron laser (XFEL) sources and the development of its synchrotron counterpart, serial synchrotron crystallography (SSX), has furthered the reach of macromolecular crystallography (MX) in time-resolved sciences and the study of protein dynamics. Although continuous efforts are being made to adapt reaction-triggering technologies, such as substrate mixing (Olmos *et al.*, 2018 ▸; Mehrabi, Schulz, Agthe *et al.*, 2019 ▸), optical laser pumping of a light-absorbing molecule remains the default choice for the vast majority of pump–probe experiments. This is unsurprising given the SFX community’s ongoing focus on light-sensitive proteins and/or ligands (Brändén & Neutze, 2021 ▸). However, continued efforts have been made to find general adaptations to expand the scope of laser pump–probe methods beyond intrinsically light-sensitive proteins. These include the laser-triggered release of photocages (Schulz *et al.*, 2022 ▸), biological photoswitches (Wranik *et al.*, 2023 ▸) and temperature jump induction in non-photoactive targets (Wolff *et al.*, 2023 ▸).

The very high peak brilliance of XFEL sources spurred the ‘diffraction before destruction’ approach methodology (Neutze *et al.*, 2000 ▸; Chapman *et al.*, 2011 ▸; Boutet *et al.*, 2012 ▸) and created the need for rapid and efficient sample-delivery systems (also beneficial in SSX). The most widely used delivery systems include injectors using gas-focusing nozzles (DePonte *et al.*, 2009 ▸), electrospun liquid microjets (Sierra *et al.*, 2012 ▸) and high-viscosity extruders (HVEs), which have proved their utility for both crystals of membrane proteins grown in the lipidic cubic phase (LCP) and crystals of soluble proteins (Weierstall *et al.*, 2014 ▸; Fromme *et al.*, 2015 ▸; Nogly *et al.*, 2015 ▸; Botha *et al.*, 2015 ▸). Together these technologies have resulted in many novel insights into protein dynamics and are widely used for time-resolved SFX (TR-SFX) experiments.

The sample efficiency of jet-based delivery methods in SFX measurements is dependent upon the repetition rate of the source, the diameter and flow rate of the jet, and the X-ray energy. The flow rates of liquid jets, such as the gas dynamic virtual nozzle (GDVN), need to be high enough to maintain a liquid filament. In a continuous jet, this is very sample consumptive at ∼10 µl min^−1^ for a jet running at 10 m s^−1^, although this can be offset by pulsing or dripping the jet in sync with the XFEL pulse (Weierstall, 2014 ▸). High sample consumption can also be mitigated by increasing the repetition rate of the source to reduce gaps between the illuminated parts of the jet (Gisriel *et al.*, 2019 ▸; Grünbein *et al.*, 2018 ▸; Wiedorn *et al.*, 2018 ▸). Using a 100 kHz source, compared with a 100 Hz source, would reduce the distance between adjacent pulses within the jet from 100 to 0.1 mm.

HVEs operate at flow rates and jet speeds more suited to the majority of low repetition rate (>1 kHz) sources currently available (as of the time of writing, May 2024). The development of the HVE significantly increased sample efficiency compared with GDVNs, as stable filaments can be maintained with jet speeds in the hundreds of micrometres per second. This translates to sample usage rates below 0.01 µl min^−1^. However, despite this improvement for low-repetition-rate sources, sample consumption for HVEs could still be improved, as part of the injected sample is still not probed by the X-ray beam.

This sample efficiency is further reduced when performing pump–probe measurements. During a pump–probe experiment, the jet speed typically has to be increased to refresh the sample within the laser-excitation area to preclude shot-to-shot light contamination. This increase is due to the larger beam profile of the pump laser with respect to the X-rays, and the distance between adjacent XFEL shots needs to be safely larger than the width of the pump pulse (Smolentsev *et al.*, 2013 ▸). Therefore, a trade-off has to be found between crystal concentration, repetition rate, and the jet width and velocities, to ensure that the sample is not overly wasted and that the experiment is properly performed. A reduction of crystal concentration and/or XFEL repetition rate will negatively impact the beam-time efficiency. Preparations for experiments often require months of work invested in purifying and crystallizing sufficient protein quantities. Therefore, any improvement in sample and beam-time efficiency is of great interest to the structural biology field.

An alternative to jet-based systems is a fixed-target delivery system where protein micro-crystals are immobilized on a solid support or chip (Zarrine-Afsar *et al.*, 2012 ▸). These techniques emerged later than their jet-based cousins but have proved to be a robust and user-friendly sample-delivery system. Fixed targets were first applied to SFX at LCLS, where a micro-crystal slurry was applied to a silicon nitride membrane and rastered through in step with the XFEL pulse (Hunter *et al.*, 2014 ▸). Since then, a large variety of different supports have emerged. These can be roughly grouped by whether or not they have apertures and whether those apertures are aligned to the beam during data collection (Carrillo *et al.*, 2023 ▸). For these aperture-aligned fixed targets, the key development was the coupling of the precision silicon fabrication technique (Oghbaey *et al.*, 2016 ▸; Mueller *et al.*, 2015 ▸) to stage motion and alignment strategies (Sherrell *et al.*, 2015 ▸). The three prominent examples of these apertured targets are the HARE (‘hit and return’) chip (Mehrabi *et al.*, 2020 ▸), the Oxford chip (Ebrahim *et al.*, 2019 ▸) and the micro-structured polymer (MISP) chip (Carrillo *et al.*, 2023 ▸). Examples of the raster-based targets are the nylon mesh and enclosed film (NAM)-based sample holder (Park *et al.*, 2020 ▸; Nam *et al.*, 2021 ▸), the advanced lightweight encapsulator for crystallography (ALEX) mesh holder (Sherrell *et al.*, 2022 ▸), and the MPI sheet-on-sheet (SOS) chip (Doak *et al.*, 2018 ▸). Solid supports have typically shown a decrease in sample use compared with the jets, without the need for gas flow or electrodes (Hunter *et al.*, 2014 ▸; Mueller *et al.*, 2015 ▸; Doak *et al.*, 2018 ▸). Given the utility of the fixed targets and optical lasers for time-resolved crystallography, chip developers must ensure that their tools are compatible with pump–probe experiments using optical lasers.

**Figure 1:**
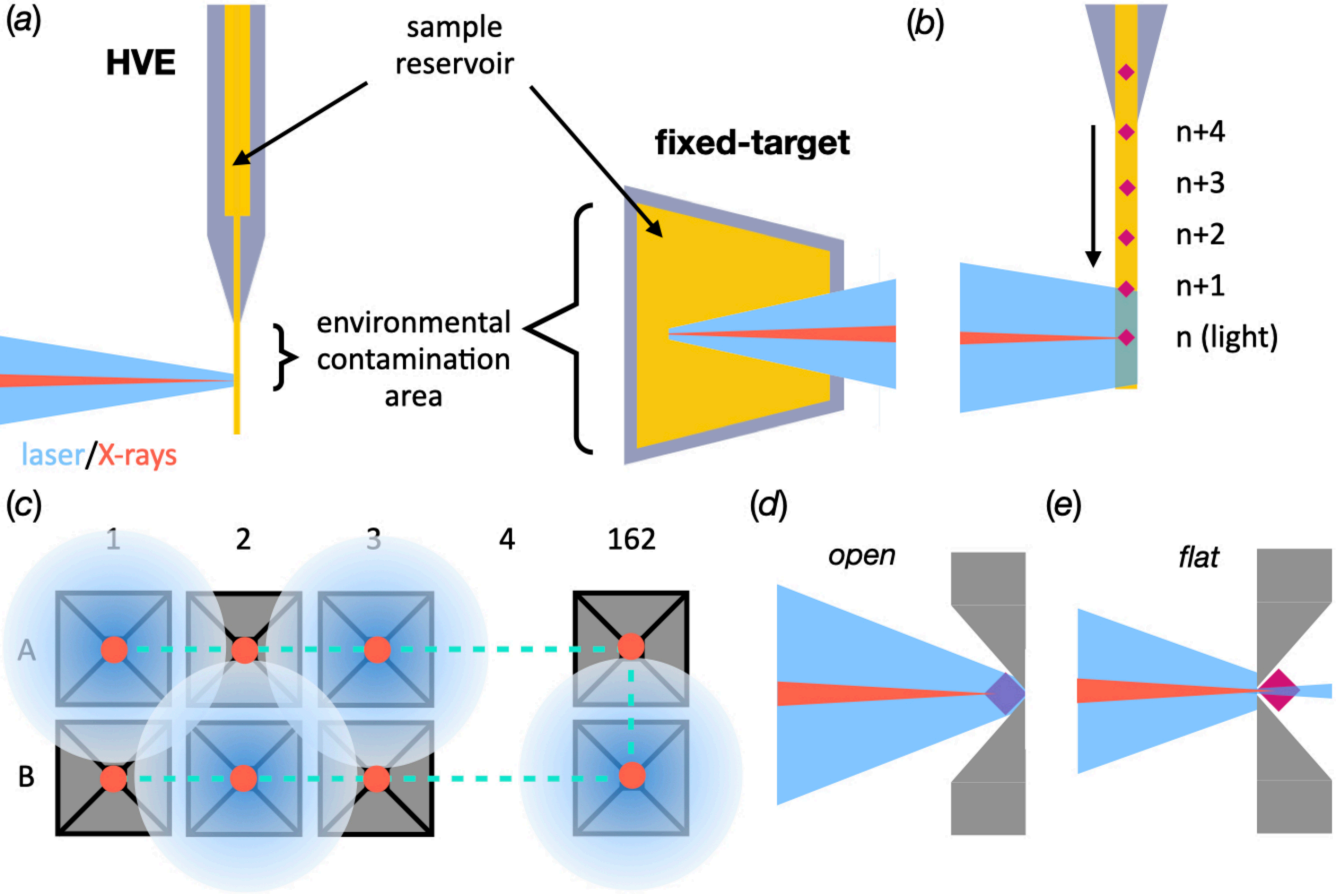
Practical differences between potential regions of light contamination in fixed targets and HVEs. (*a*) A comparison of pump–probe in HVEs and fixed targets, highlighting the increased access of environmental stray photons to the sample when mounted in fixed targets compared with jets. (*b*) A simplified schematic drawing demonstrating pump-laser contamination in an HVE. Crystal *n* is currently illuminated with X-rays. As the jet descends, crystals *n* + 1, *n* + 2, *etc.*, will descend into the X-ray path. The pump laser is also currently illuminating *n*, but is also partially illuminating *n* + 1 and leading to light contamination. (*c*) A schematic drawing of a 1:1 interleaved light:dark setup in a MISP chip with an incompatibly large laser spot for the experiment. Each ‘light well’ (A1, A3, A5, *etc.*) is correctly illuminated. The laser will also weakly illuminate any crystals in the ‘dark wells’ (A2, A4, A6, *etc.*) and lead to light contamination. Importantly, however, the light contamination will occur between both consecutive apertures (A1 to A2, 10 ms at 100 Hz) and adjacent apertures, *i.e.* A1 to B1. The contamination of these adjacent apertures gives rise to much longer reaction timescales; A1 to B1 = 3.24 s. (*d*), (*e*) Schematic drawings of the two potential orientations of an apertured fixed target in a pump–probe experiment. The sample area is shown in yellow, the cavities in grey and the X-ray in red. The stage path is depicted as a dashed cyan line and the pump laser is shown in blue.

As for all pump–probe sample-delivery systems, a significant issue when using fixed targets is how to ensure that samples remain free from light prior to laser illumination and that only the desired crystals are illuminated at the designated moment. The former of these forms of light contamination can also be thought of as unintended pre-illumination from the environment. This environmental contamination can occur at any moment from attempting to load the sample into the delivery system and could result from a poor choice of dark-room lamps during sample loading, transporting loaded samples to the hutch or perhaps poor shielding of hutch lights/light-emitting diodes [Fig. 1 ▸(*a*)]. The latter comes from contamination of adjacent crystals with respect to the desired crystal from the optical laser pulse [Figs. 1 ▸(*b*) and 1 ▸(*c*)]. For example, data collected during a pump–probe experiment typically interleave dark and laser-pumped XFEL exposures. If the laser and delivery-system parameters are not properly characterized, significant light contamination of both the interleaved ‘light’ and ‘dark’ diffraction patterns can occur. This contamination, ultimately, prevents the correct interpretation of relevant structural changes and renders the experiment pointless.

In jet and extruder-based delivery systems, the oncoming crystals are housed in the opaque enclosure of the reservoir. Environmental pre-illumination is a concern during sample preparation and loading, but, once complete, this risk is minimized due to the small area of environment exposure during data collection [Fig. 1 ▸(*a*)]. Nevertheless, adjacent-crystal contamination is still a significant concern. However, contamination, if it does occur, will be limited to the single axis of the jet and will likely be restricted to the immediately following shot(s) [Fig. 1 ▸(*b*)].

Unfortunately, both of these risks are amplified when using fixed targets. Firstly, the whole chip is left exposed to its surroundings and, therefore, the risk of environmental contamination is increased [Fig. 1 ▸(*a*)]. Pump-laser contamination in fixed targets is also more pervasive than in the jet since the adjacent crystals are distributed in two dimensions. This means that light contamination can propagate much further in time from the deliberately pumped crystal [Fig. 1 ▸(*c*)]. This could be tens of milliseconds or even seconds depending on the location of contamination and the repetition rate used for data collection. This uncertainty in the effective time delay of the probed region can ultimately make any intelligent subsequent analysis very challenging or, most likely, impossible.

XFELs are uniquely suited to the study of ultrafast dynamics on the femtosecond–microsecond scale; however, fixed targets have yet to be exploited for time-resolved work at XFELs, although their utility has been proven for similar experiments at synchrotrons (Mehrabi, Schulz, Dsouza *et al.*, 2019 ▸). The serial crystallography with solid-support MX (SwissMX) endstation at the SwissFEL Cristallina experimental station (Paul Scherrer Institute, Villigen, Switzerland) and PSI-developed MISP chips were designed to address this gap. However, extensive tests are obviously essential to validate these tools for optical pump X-ray probe experiments. This work describes a commissioning experiment conducted in November 2022 at the SwissFEL Cristallina experimental station. The aim was to find and understand the parameters that enable time-resolved pump–probe experiments using the SwissMX and MISP chips (Carrillo *et al.*, 2022 ▸). To achieve this, a pump–probe experiment was performed collecting interleaved dark (no laser) and light (laser on) diffraction images (hereafter referred to as interleaved dark and interleaved light, respectively) in a 1:1 ratio with both transparent and opaque MISP chips. The protein used in these experiments was the light–oxygen–voltage (LOV) domain 1 from *Chlamydomonas reinhardtii* (*Cr*LOV1). *Cr*LOV1 is a light-sensitive protein domain that undergoes a long photocycle (∼200 s cycle time). This long photocycle is, therefore, ideal for the purpose of investigating light contamination in fixed targets where any unsolicited light originating from stray photons from adjacent cavities can occur on the millisecond to tens of seconds scales. A covalent thio­ether bond forms between the flavin mononucleotide (FMN) cofactor and Cys57 in the flavin binding pocket after photoexcitation (Fedorov *et al.*, 2003 ▸). This distinctive signature makes *Cr*LOV1 an ideal candidate to benchmark data-collection schemes using fixed targets with respect to light contamination.

## 2. Materials and methods

### 2.1. Expression and purification

The expression and purification protocols have been described previously (Gotthard *et al.*, 2023 ▸). Briefly, the expressed protein sequence was amino acids 16–133 of the Phot1 protein LOV1 domain from *C. reinhardtii*. The protein possesses an N-terminal His tag followed by a factor Xa protease site. The protein was expressed in *Escherichia coli* BL21 DE3 using auto-inducible media (Studier, 2005 ▸). The expressed protein was purified by Ni^2+^ affinity followed by size-exclusion chromatography. Fractions corresponding to the protein were pooled and concentrated to 10 mg ml^−1^ for crystallization.

### 2.2. Crystallization

Limited proteolysis with trypsin allowed a more reproducible and controlled crystallization process (Gotthard *et al.*, 2023 ▸). Microcrystals were grown at 293 K in batch with a 2:1 protein-to-precipitant condition ratio in 0.1 *M* sodium cacodylate pH 6.5, 1.0 *M* sodium citrate dibasic trihydrate. An average crystal size of 25 µm ∓ 7 µm was measured using a Leica microscope (Kaminski *et al.*, 2022 ▸). The crystal concentration was measured using a cell counter and estimated to be 1.0–2.0 × 10^7^ crystals ml^−1^.

### 2.3. Chip mounting

Firstly, the crystal concentration was adjusted by diluting the microcrystal slurry with crystallization solution to a final concentration of 1–2 × 10^6^ crystals ml^−1^. Subsequently, 400 µl of crystalline suspension was pipetted onto a MISP chip that was made either with transparent cyclic olefin polymer (COP) film or with an opaque film made by mixing 10%(*w*/*w*) carbon black with cyclic olefin copolymer (COC) (Carrillo *et al.*, 2023 ▸). Once loaded, the MISP chip was placed on a loading stage connected to a vacuum pump that served to remove the excess mother liquor and funnel the crystals into the wells. Filter paper was occasionally required to blot away any excess solution. After this, the chip was placed onto MISP-chip holders (Carrillo *et al.*, 2023 ▸) that sealed the chip inside two pieces of 6 µm Mylar film and maintained the crystal hydration. This was then placed inside a darkened humidity chamber kept at 80% relative humidity and transported to the beamline. X-ray data collection was performed at the Cristallina experimental station of the SwissFEL using the SwissMX endstation. Due to concerns over crystal hydration, only five chips were consecutively loaded and kept in the humidity chamber at a time. Chips were manually mounted from the humidified chamber to SwissMX.

### 2.4. Beamline setup and data collection

Data were collected over a 24 h period on 27 November 2022. The X-ray beam energy was 12.4 keV with a pulse energy at the sample position of ∼50 µJ. The X-rays were focused using Kirkpatrick–Baez mirrors to a spot size of ∼1.5 × 1.5 µm and the repetition rate was 100 Hz. The pulse width was ∼35 fs root mean square. The diffraction data were recorded on a JUNGFRAU 8 Mpixel detector. Collecting each set of five chips stored in the humidity chamber took ∼50–60 min. The chips were kept at room temperature (296–298 K, depending on the location within SwissFEL).

### 2.5. Laser coupling

As of November 2022, the Cristallina hutch was not provided with a through-space connection to a pump laser. The SwissMX endstation was limited to a 70 m fibre connection to a nanosecond laser located in the SwissFEL laser room. Due to this constraint, the decision was taken to couple the laser to the sample position through the SwissMX on-axis-viewing (OAV) system. Such couplings have been demonstrated at synchrotron protein crystallography beamlines (Pompidor *et al.*, 2013 ▸; Madden *et al.*, 2013 ▸), and this solution offered the best compromise between the final achievable focus and the meshing with other instrumentation at the sample position.

For pump–probe experiments using fixed targets for sample delivery, the key parameter for avoiding light contamination of unprobed crystals by the pump laser is the laser focal-spot size. The efficiency of the fixed target is dependent upon the number of crystal locations that can be squeezed onto the chip surface. For the apertured fixed targets, this means having a small pitch between adjacent cavities. The achievable laser focus size, therefore, has a direct influence on the efficiency of the experiment, as an increase in the diameter of the focus will necessitate an increase in the aperture pitch or in the spacing of the XFEL probed cavities.

The laser spot size was estimated to be 50 × 50 µm based on its reflection from a piece of opaque COC film held at the OAV focus. The OAV system was calibrated against known distances, but it was impossible to accurately infer from this the 1/e^2^ or full width at half-maximum of the spot profile. The laser wavelength was set at 450 nm with a pulse energy of ∼2 µJ at the sample position. The duration of the laser pulse was 3 ns (FWHM) and the laser was not polarized due to the fibre coupling. Given the average crystal dimensions of 25 × 25 × 25 µm, the ɛ_450nm_ for the flavin was estimated to be 11 300 *M*^−1^ cm^−1^ for the free flavin. Since the beam profile could not be visualized, it was not possible to determine whether the beam profile was more top-hat or Gaussian. Given this ambiguity, we estimate the mean number of photons per chromophore to be between 1.6 and 3.2 (Grünbein *et al.*, 2020 ▸).

### 2.6. Pump–probe setup

X-ray data collection over the whole chip in a dark environment without any laser excitation was used as a ‘reference’ for the calculation of the Fourier difference electron-density maps (*F*_obs_^laser-off^). Light contamination from the transparent and opaque chips and the SwissMX setup was assessed using two chip orientations: ‘open’, with the wider side of the cavity directed towards the laser, and ‘flat’, with the wider side of the cavity facing the detector. All pump–probe data were collected at a 1:1 ratio of interleaved light:dark, *i.e.* XFEL at 100 Hz, nanosecond laser at 50 Hz, giving a laser pulse in every other aperture.

### 2.7. Data processing

Serial data processing was performed using the *CrystFEL* version 0.10.1 software suite (White, 2019 ▸). Diffraction hits were identified using the *peakfinder8* and *XGANDALF* (Gevorkov *et al.*, 2019 ▸) algorithms with a hexagonal unit cell (*a* = *b* = 122, *c* = 46 Å). Peak integration was performed using the three-rings methods in *indexamajig* with integration radii of 2, 3 and 5 pixels. Indexing rates were between 50 and 80%. The interleaved-dark and interleaved-light image lists were generated by labelling images with a ‘laser-on’ event generated by the SwissFEL event master. This event was passed to the JUNGFRAU detector while data were collected, and propagated with it thereafter. By following the laser events, the interleaved data could be indexed and integrated independently. Stream files were merged using *partialator* using the unity partiality model with a pushres option of 1.6–2.0 nm^−1^. Furthermore, *hkl* files were converted into the mtz format with *f2mtz* from the *CCP*4 suite (Winn *et al.*, 2011 ▸). A resolution cut-off was applied when CC_1/2_ was falling below 30%. Dataset statistics are reported in Table 1 ▸.

### 2.8. Isomorphous difference maps

Fourier difference electron-density maps were calculated using the phenix.fobs_minus_fobs_map program from the *Phenix* suite (Liebschner *et al.*, 2019 ▸). A resolution cut-off of 1.8 Å and a sigma cut-off of 3.0 were applied, and the multiscale option was used to calculate the difference maps. The presence of contamination was observed by subtracting data collected without laser illumination (laser off) from the interleaved data (interleaved dark or light): *F*_obs_^interleaved-dark-or-light^ − *F*_obs_^laser-off^. Assuming that no contamination can be observed, the signal from *F*_obs_^interleaved-light^ − *F*_obs_^laser-off^ should also be the same as the interleaved difference map, *F*_obs_^interleaved-light^ − *F*_obs_^interleaved-dark^. Figures were prepared using *PyMOL* (DeLano, 2002 ▸).

## 3. Results and discussion

### 3.1. Pump–probe with fixed targets

**Figure 2:**
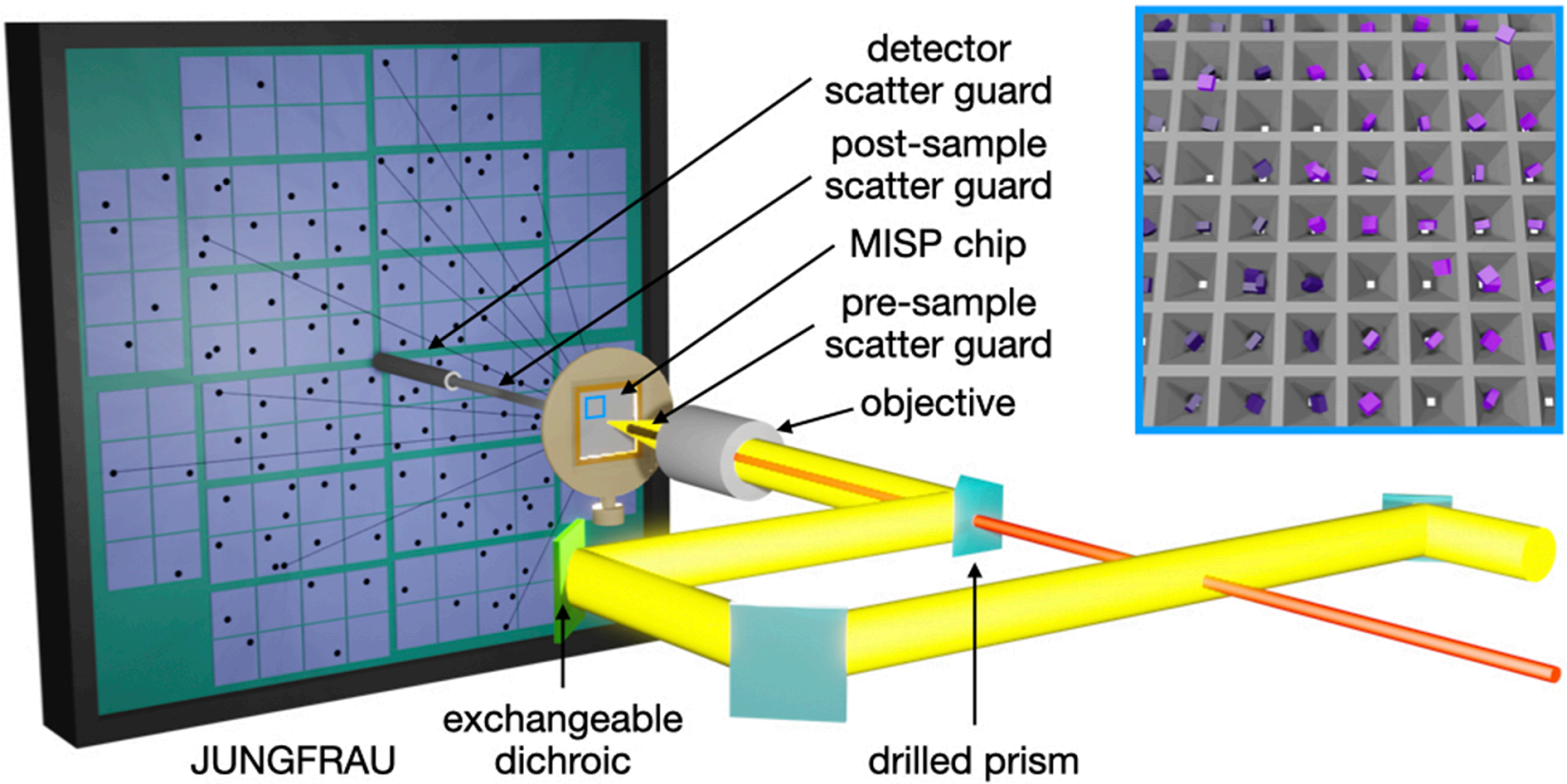
A schematic drawing of the pump–probe experimental setup at the SwissMX endstation at the SwissFEL Cristallina experimental station. The pump laser (yellow) is coupled to the sample position via the endstation’s OAV system. An exchangeable dichroic mirror (light green) enables different pump wavelengths to be reflected whilst transmitting light for the chip alignment into a camera (not shown in this diagram). The X-rays pass through the centre of the drilled objective and prism of the final part of the laser coupling. Air scatter from the X-rays is minimized using pre- and post-sample scatter guards. The blue box highlights an area of the chip showing a 1:1 interleaved light:dark scheme, where X-rays are delivered to every well and laser pumps only to every other.

Here, we present laser-triggered pump–probe experiments using the SwissMX endstation and MISP chips at the SwissFEL Cristallina experimental station. To translate samples, SwissMX is equipped with two orthogonal linear stages (Parker), for *x* and *y* motions, and two additional stages of *z* and *x* motion (Standa). The MISP chips have a reliable active area of 162 × 162 pyramidal cavities totalling 26 244 apertures per chip. All data from the chips were collected in a serpentine-like pattern. Fig. 2 ▸ shows the final stage of the optical pump-laser coupling through the SwissMX OAV system. The setup is currently only used for in-air data collection. Therefore, scatter guards are required to catch the beam from the OAV system to the sample and from the sample to the detector face. The total exposed length to air is ∼15 mm.

Data were collected on two different chip types, named ‘transparent’ and ‘opaque’ from their transparency to the visible spectrum or lack thereof, and in two different orientations, ‘open’ and ‘flat’. The transparent chips were made from commercially available 50 µm COP film, whereas the opaque chips were fabricated with an in-house cast film using COC pellets and the addition of carbon black powder (Carrillo *et al.*, 2023 ▸)

**Figure 3:**
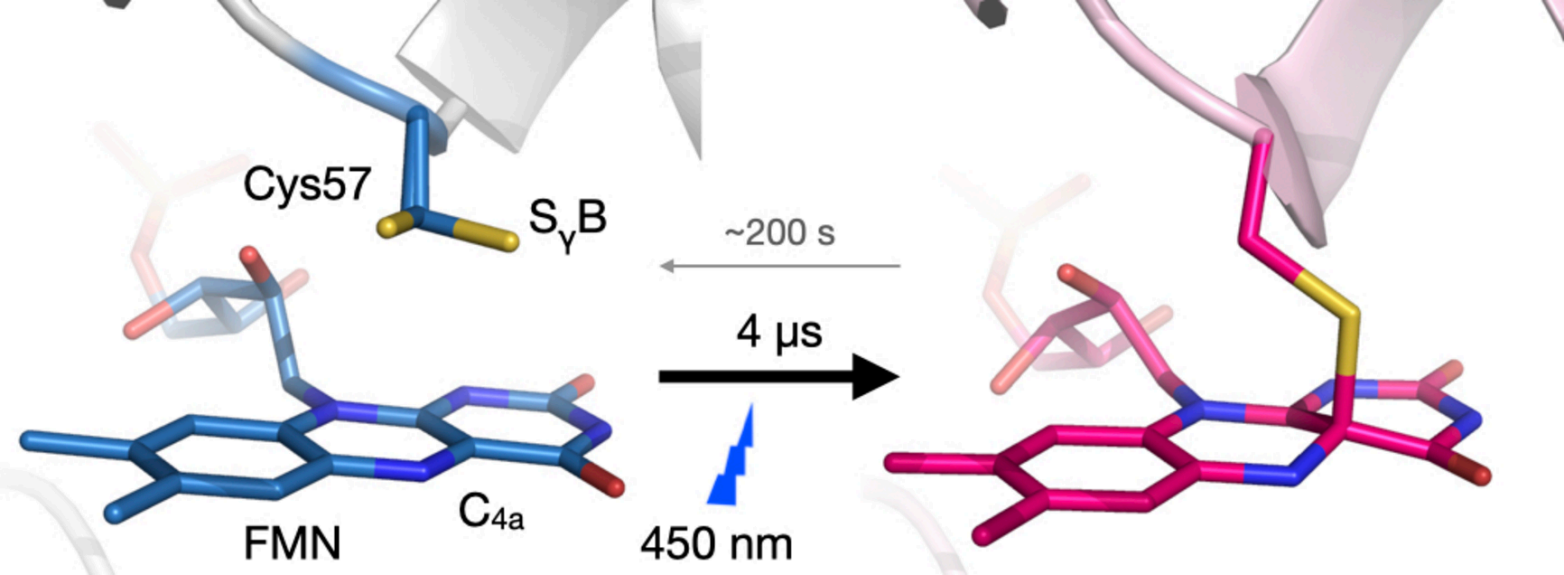
Thio­adduct formation between *Cr*LOV1 and its FMN cofactor upon illumination. Crystal structures of the *C. reinhardtii* LOV1 domain in light and dark stationary states present two conformations of the binding-site cysteine (Cys57) (Fedorov *et al.*, 2003 ▸). Blue-light absorption causes the formation of a covalent bond between the flavin C(4a) and the thiol of Cys57 within 10 µs. The reaction proceeds through an excited flavin singlet to a triplet state that then decays monotonically to the adduct (Holzer *et al.*, 2002 ▸; Kottke *et al.*, 2003 ▸), in which this cysteine moves ∼1.5 Å closer to the FMN-C(4a) for adduct formation (Fedorov *et al.*, 2003 ▸). Thio-adduct formation then triggers rearrangements throughout the whole LOV domain (Gotthard *et al.*, 2023 ▸).

Time-resolved spectroscopy experiments on the *Cr*LOV1 used in this work indicate that it undergoes the formation of a covalent thio­ether bond between its FMN cofactor and Cys57 10 µs after photoexcitation (Kottke *et al.*, 2003 ▸; Holzer *et al.*, 2002 ▸), which then persists late into the photocycle (Fedorov *et al.*, 2003 ▸; Kottke *et al.*, 2003 ▸) (Fig. 3 ▸). Given the significant strength of the expected signal in the Fourier difference maps for the covalent bond formation, a 10 µs time delay was selected to test the suitability of the *Cr*LOV1 crystals for TR-SFX experiments and to make use of their long (∼200 s) photocycle to serve as a light-contamination indicator.

We employed a 1:1 interleaved light:dark experimental routine with the XFEL at 100 Hz and the pump laser at 50 Hz. While a comprehensive analysis of the LOV-FMN light-activated structure is beyond the scope of this article, it suffices to expect a photoactivated structure with the FMN–Cys covalent adduct formation in the laser-exposed crystals. The unpumped crystals should yield a dark-state structure without the thio­ether bond signature. We also collected SFX data entirely without the pump laser (laser off) for reference as a ‘properly’ dark state. Ideally, the laser-off should be indistinguishable from the dark datasets in the interleaving TR-SFX experiment.

It is key to stress the importance of delivering light-contamination-free time-resolved pump–probe data. Otherwise, the crystallographic data would represent multiple overlapping protein trajectories triggered by more than one pump-laser pulse, impairing correct interpretation of the electron densities. Importantly for the current work, the *Cr*LOV1 domain was specifically chosen for the fixed-target pump–probe commissioning since the 200 s resting-state recovery time of the domain significantly exceeds the 10 ms interval between the consecutive XFEL pulses into the adjacent cavities of the chip. The 200 s recovery time also exceeds the time to image over half the chip, so potential light contamination in adjacent columns will also be observed.

In the quest to explore different experimental geometries of the fixed-target setup, two orientations of the opaque chips were tested with respect to the incidence pump laser and X-rays. One orientation where the chip was mounted with the pyramidal cavity facing towards the X-ray and optical laser beams (open side) and one in the opposite direction (flat side) [Figs. 1 ▸(*d*) and 1 ▸(*e*)]. The transparent chip was only tested in one orientation (open).

### 3.2. Assessment of contamination

**Figure 4:**
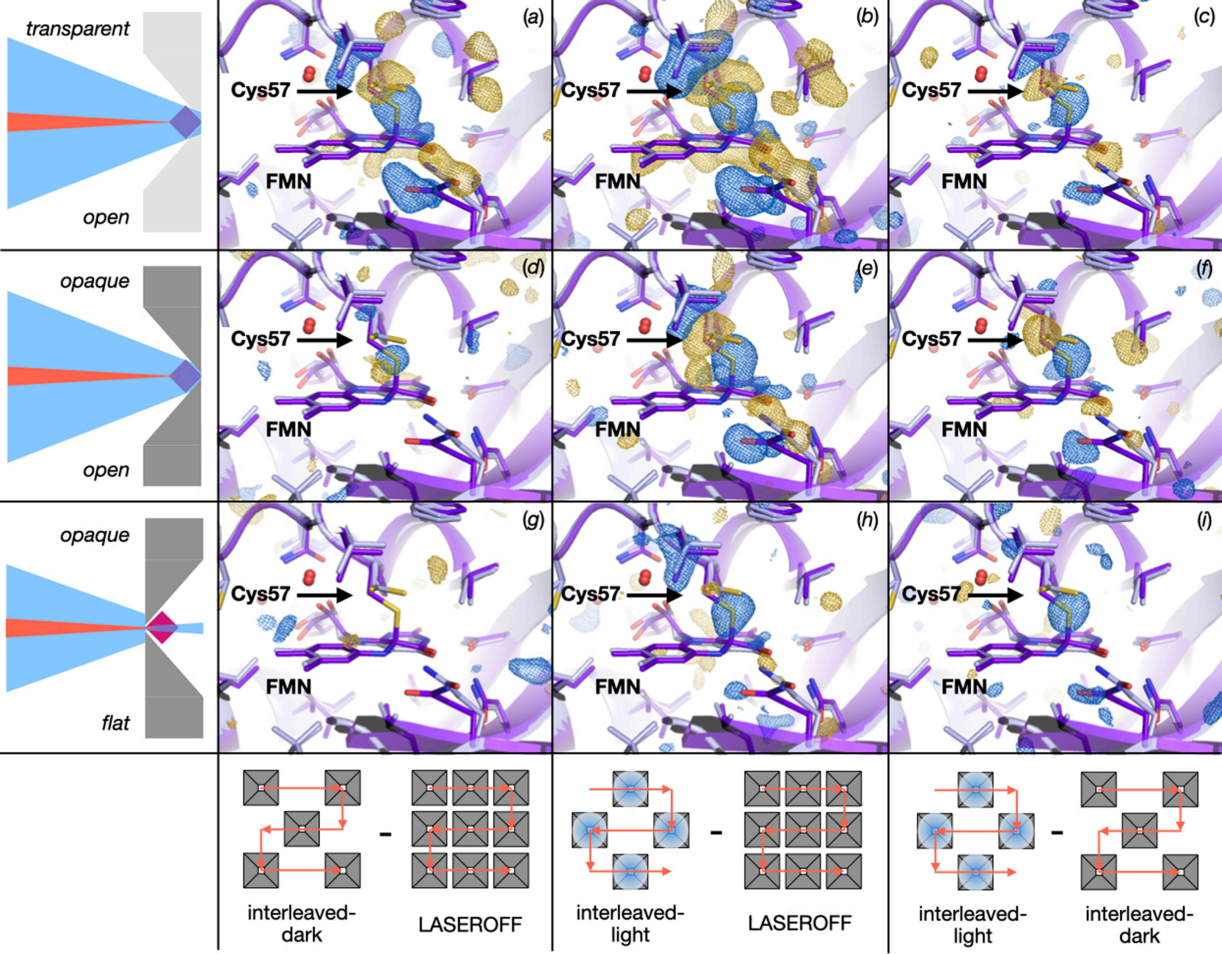
Chip placement for laser incidence and Fourier difference electron-density maps for TR-SFX with *Cr*LOV1 at Δ*t* = 10 µs. Three different setups were investigated. Shown in the upper row is a transparent chip with cavity facing the pump pulse. This geometry yields an activation signal in all three maps, corresponding to thio­ether bond formation between the active-site cysteine and FMN: (*a*) *F*_obs_^interleaved-dark^ − *F*_obs_^laser-off^, (*b*) *F*_obs_^interleaved-light^ − *F*_obs_^laser-off^ and (*c*) *F*_obs_^interleaved-light^ − *F*_obs_^interleaved-dark^. The middle row shows that data collection using the opaque chip with cavity facing the pump pulse yields an activation signal in all three maps: (*d*) *F*_obs_^interleaved-dark^ − *F*_obs_^laser-off^, (*e*) *F*_obs_^interleaved-light^ − *F*_obs_^laser-off^ and (*f*) *F*_obs_^interleaved-light^ − *F*_obs_^interleaved-dark^. The bottom row shows that data acquisition using the opaque chip with aperture facing the pump pulse yields an activation signal where expected, while the lack of signal in the *F*_obs_^interleaved-dark^ − *F*_obs_^laser-off^ map indicates that light contamination was avoided in this setup: (*g*) *F*_obs_^interleaved-dark^ − *F*_obs_^laser-off^, (*h*) *F*_obs_^interleaved-light^ − *F*_obs_^laser-off^ and (*i*) *F*_obs_^interleaved-light^ − *F*_obs_^interleaved-dark^. The Fourier difference electron-density maps show positive and negative densities as blue and gold mesh, respectively, highlighting differences between datasets. The cartoon and sticks representation shows in light grey the dark state of the protein with its FMN ligand adjacent to but not covalently bound to Cys57. As a reference, the photoactivated late photocycle intermediate with FMN covalently bound to Cys57 is shown in purple. A schematic representation of the chip (grey) with a crystal sample (pink/blue) placement with respect to pump laser (blue) and XFEL pulses (red) is shown at the very left. The bottom panel shows the practical arrangement of the data from the wells contributing to the different maps.

The TR-SFX experiment was carried out with a Δ*t* = 10 µs between the laser pump and X-ray probe pulses. Fourier difference electron-density maps were evaluated in terms of photoactivation yield and potential light contamination in the nearby wells. The transparent-chip setup yielded a positive signal indicating thio­ether bond formation in *F*_obs_^interleaved-dark^ − *F*_obs_^laser-off^ [Fig. 4 ▸(*a*)], *F*_obs_^interleaved-light^ − *F*_obs_^laser-off^ [Fig. 4 ▸(*b*)] and *F*_obs_^interleaved-light^ − *F*_obs_^interleaved-dark^ [Fig. 4 ▸(*c*)]. While it shows features characteristic of the light-activated state, the light signal present in the *F*_obs_^interleaved-dark^ − *F*_obs_^laser-off^ map indicates that each pump laser pulse did not only photoactivate a single well of the chip but also that the light reached the adjacent wells leading to light contamination. Although light contamination was more likely in the transparent chips, the extent of its prevalence was not expected. Interestingly, the signal for *F*_obs_^interleaved-dark^ − *F*_obs_^laser-off^ was as strong as for the *F*_obs_^interleaved-light^ − *F*_obs_^interleaved-dark^ maps. The light contamination was likely due to the transmission of the laser light orthogonally through the chip from the scattering source, either via interaction with the crystal, chip or both.

Next, we performed tests using the opaque MISP chips in the two orientations (open and flat). The open orientation, which allows the most light to reach the sample and maximizes the excited fraction of molecules, again yielded a positive signal, indicating the thio­ether bond formation in the *F*_obs_^interleaved-dark^ − *F*_obs_^laser-off^ map [Fig. 4 ▸(*d*)]. Despite yielding significantly lower signals than in the case of the transparent chips, contamination was still evident. Qualitatively similar signal was also present in the *F*_obs_^interleaved-light^ − *F*_obs_^laser-off^ [Fig. 4 ▸(*e*)] and *F*_obs_^interleaved-light^ − *F*_obs_^interleaved-dark^ maps [Fig. 4 ▸(*f*)].

However, in the alternative chip orientation, with the flat side of the cavity now facing the pump laser, no thio­ether signal was observed in the *F*_obs_^interleaved-dark^ − *F*_obs_^laser-off^ map [Fig. 4 ▸(*g*)]. This lack of thio­ether bond signal shows that there was no detectable light contamination. At the same time, the *F*_obs_^interleaved-light^ − *F*_obs_^laser-off^ [Fig. 4 ▸(*h*)] and *F*_obs_^interleaved-light^ − *F*_obs_^interleaved-dark^ [Fig. 4 ▸(*i*)] maps both show the expected electron-density signature for the covalent adduct formation. Therefore, the opaque chips with flat-side incidence of the laser light onto the sample did prove to be a successful setup in our experiment.

**Figure 5:**
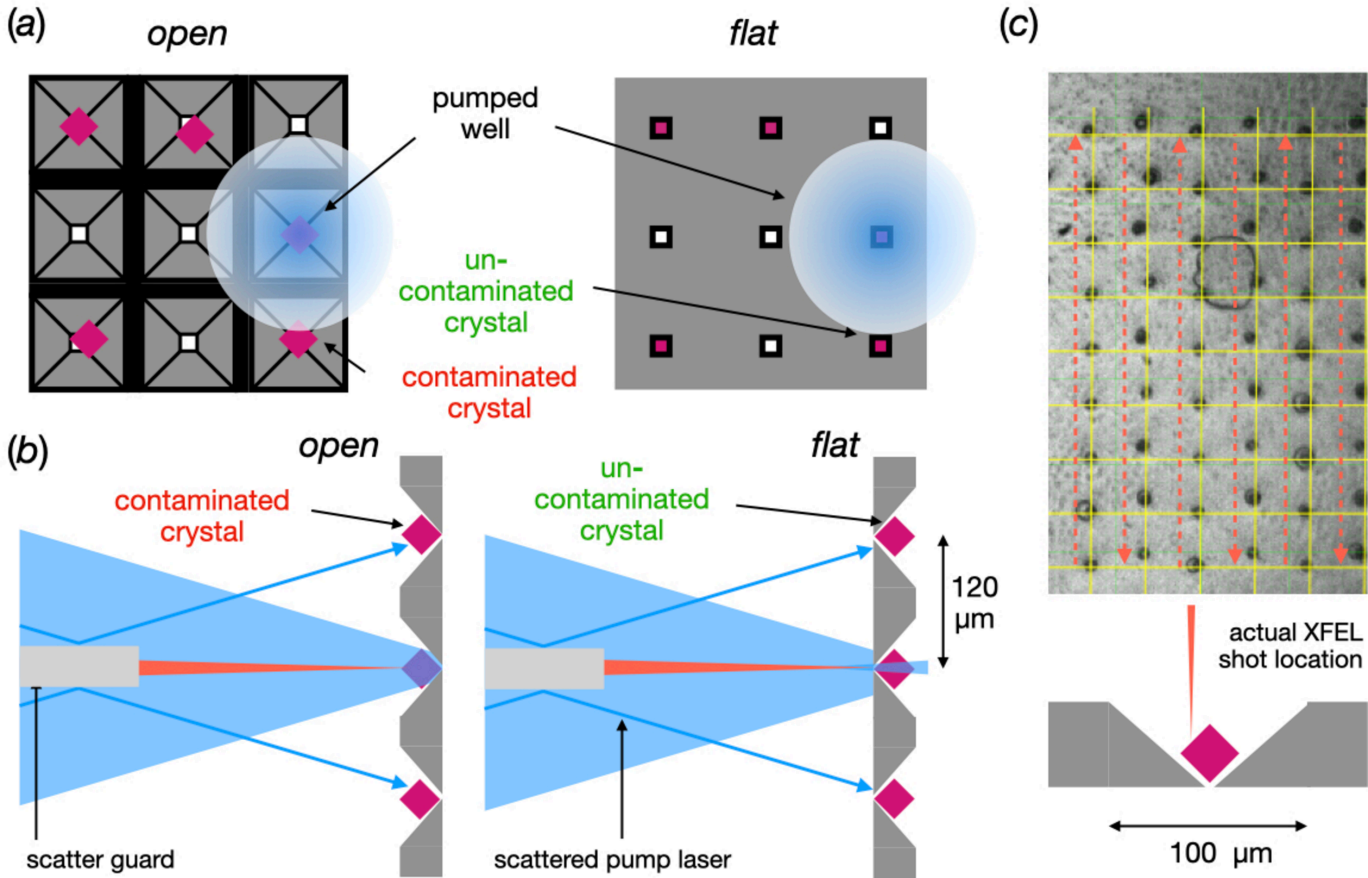
Possible explanations for the light contamination being observed in the open orientation but not in the flat orientation. (*a*) A schematic drawing of the X-ray/pump-laser view of the chip in the open and flat orientations. In the open view, the entire crystal is visible, enabling contamination via a large laser profile. The same large laser profile in the flat orientation does not give rise to contamination due to the restricted view of the crystals. (*b*) Schematic drawings showing how potential scattered pump laser from the pre-sample scatter guard could give rise to light contamination. Again, the restricted view of the crystals in the flat orientation prevents contamination of crystals in adjacent wells. (*c*) An image collected with the help of the Max Planck Institute, Heidelberg using an SOS chip (Doak *et al.*, 2018 ▸) containing 1 *M* cadmium chloride and a schematic drawing of its implications for the MISP chip. The image was collected using the OAV camera of SwissMX. The yellow grid shows the intended shot locations. The red arrow shows the direction of travel of the stages. Due to the 1 ms offset of the stage motion and XFEL, the XFEL shot is 12 µm off the aperture centre.

The reason for the success of the experiment in the flat orientation compared with the open orientation was likely due to the reduced potential exposure of the crystals in this orientation (Fig. 5 ▸). As stated in *Materials and methods*[Sec sec2], due to issues at the beginning of beam time, the laser beam profile could not be precisely measured, only approximately inferred from scattered light off a black film. This means that the laser profile could conceivably be larger than the 50 × 50 µm estimate. Fig. 5 ▸(*a*) shows how an increased laser profile could enable light contamination in the open chip orientation. The flat orientation could, by restricting the view of the crystals by the pump laser, help to prevent the contamination even with a larger laser profile. It is possible that stray scattered light of either the collimator or the chip sealing film may have been a contaminating factor [Fig. 5 ▸(*b*)]. Another potential contributing factor was the synchronization of the stage motion and the XFEL pulse. Subsequent experiments have shown that the XFEL pulse was delivered ∼1 ms (12 µm) behind its intended location, *i.e.* the XFEL pulse was not hitting the centre of each aperture but 12 µm off on the side of the aperture [Fig. 5 ▸(*c*)].

### 3.3. Reduction in sample consumption

Sample preparation is a laborious and challenging task for TR-SFX experiments. Various sample parameters must be considered and optimized depending on the planned experiment and delivery system used. Not only does the sample need to be of high quality but the large quantities of crystalline protein can also require months of sample production in preparation for every experiment. One of the attractive features of fixed targets is their low sample consumption when compared with other delivery methods. The sample consumption from our fixed-target TR-SFX experiment was calculated to be only ∼200 µg for the collection of 10 000 indexed lattices. For comparison, Table 2 ▸ shows the quantities of samples consumed in several jet-based experiments with 120 Hz and lower X-ray pulse repetition rates. The development of the HVE for sample delivery was a significant improvement (>10×) when it comes to sample consumption compared with the first GDVN experiments, and has now been responsible for a considerable number of successful TR-SFX experiments (Nogly *et al.*, 2018 ▸, 2016 ▸; Mous *et al.*, 2022 ▸; Nango *et al.*, 2016 ▸; Suga *et al.*, 2017 ▸; Skopintsev *et al.*, 2020 ▸; Claesson *et al.*, 2020 ▸; Nass Kovacs *et al.*, 2019 ▸; James *et al.*, 2019 ▸; Hosaka *et al.*, 2022 ▸; Liu *et al.*, 2022 ▸; Maestre-Reyna *et al.*, 2022 ▸; Li *et al.*, 2021 ▸). However, as noted in *Introduction*[Sec sec1], experiments at high pulse repetition rate XFEL facilities should significantly reduce sample consumption with GDVN delivery (Pandey *et al.*, 2020 ▸).

The use of the MISP chip reported here required approximately ten times less sample than HVEs, with less than 1 mg of protein required for a full dataset. With every improvement in sample delivery, TR-SFX becomes more accessible to a larger scientific community and a wider range of interesting targets, many of which may not be easily overexpressed in greater quantities. Another practical point is that the preparation for the experiments can now be shortened in many cases from months to weeks, facilitating timely completion of the TR-SFX projects.

A drawback of sample delivery using the MISP chips is the time that it takes for loading and mounting each chip in a humid environment and for transporting the chips in a humidity chamber from the dark room to the beamline, a procedure that took around half an hour for five mounted chips. In addition, light contamination was an important concern when compared with jet-based experiments, where the sample is enclosed and stored in a dark reservoir until it is finally injected in a stream. The MISP chips, and other patterned fixed targets, are also not obviously suitable for membrane protein crystals grown in LCP. These crystals are hard to separate from the cubic phase and fail to populate the apertures. However, moving the LPC-grown crystals into the much less viscous sponge phase could provide a valuable alternative. Our commissioning experiment shows that light-contamination-free SFX and TR-SFX data can still be acquired using the flat orientation of the chip. Furthermore, there is also the potential to employ robotic systems for the chip mounting that will undoubtedly increase the efficiency of beam-time usage.

## 4. Conclusions

XFELs have allowed for the rapid growth of time-resolved structural experiments, which provide crucial information on the function of biological machines and molecular mechanisms. This is the first experiment of its type using a light-activated protein *Cr*LOV1 at the Cristallina endstation of the SwissFEL and using the MISP chips for sample delivery. The light contamination present with the transparent MISP chip was interesting to observe and showed the absolute necessity for using an opaque chip. With the opaque chips, the laser spot size and various other parameters are still critical to the success of the experiment. Many of these, particularly the laser spot profile, undoubtedly lead to the contamination observed in the open orientation. Future commissioning experiments will focus on enabling TR-SFX in the open orientation. This conformation has the greater propensity for crystal excitation, as the flat orientation is very unforgiving for crystals that are not perfectly sitting inside the aperture and if the chip is slightly misaligned. The reported experiments are an important step towards making XFEL fixed-target sample delivery compatible with pump–probe time-resolved experiments and making these experiments more sample efficient. Increasing sample and experimental-time efficiency will make these experiments more attainable for the general structural biology community.

## Figures and Tables

**Table 1:** Data-collection parameters and data reduction for the first experiment

	Laser off	Interleaved dark	Interleaved light	Interleaved dark	Interleaved light	Interleaved dark	Interleaved light
Data-collection parameters							
Chip type	Transparent	Transparent	Transparent	Opaque	Opaque	Opaque	Opaque
Chip orientation	Open	Open	Open	Open	Open	Flat	Flat
Δ*t* (µs)	10	10	10	10	10	10	10
Beamline, endstation	ARAMIS, Cristallina	ARAMIS, Cristallina	ARAMIS, Cristallina	ARAMIS, Cristallina	ARAMIS, Cristallina	ARAMIS, Cristallina	ARAMIS, Cristallina
Detector	JUNGFRAU 8M	JUNGFRAU 8M	JUNGFRAU 8M	JUNGFRAU 8M	JUNGFRAU 8M	JUNGFRAU 8M	JUNGFRAU 8M
X-ray energy (keV)	12.29	12.29	12.29	12.29	12.29	12.29	12.29
Laser wavelength (nm)	450	450	450	450	450	450	450
Laser profile (µm)	∼50 × 50	∼50 × 50	∼50 × 50	∼50 × 50	∼50 × 50	∼50 × 50	∼50 × 50
Repetition rate (Hz)	100	100	100	100	100	100	100
Crystal size (µm^3^)	25	25	25	25	25	25	25
							
Data reduction							
Space group	*P*6_5_22	*P*6_5_22	*P*6_5_22	*P*6_5_22	*P*6_5_22	*P*6_5_22	*P*6_5_22
Cell dimensions *a*, *b*, *c* (Å), α, β, γ (°)	122.6, 122.6, 46.6, 90, 90, 120	122.6, 122.6, 46.6, 90, 90, 120	122.6, 122.6, 46.6, 90, 90, 120	122.6, 122.6, 46.6, 90, 90, 120	122.6, 122.6, 46.6, 90, 90, 120	122.6, 122.6, 46.6, 90, 90, 120	122.6, 122.6, 46.6, 90, 90, 120
Collected images	210760	184413	184417	171246	171239	131725	131725
Indexed lattices	151790	101483	101688	72918	72229	68637	69208
Indexing rate (%)	72.0	55.0	55.1	42.6	42.2	52.1	52.5
Resolution (Å)	37.09–1.42 (1.44–1.42)	37.11–1.47 (1.50–1.47)	37.11–1.47 (1.50–1.47)	37.12–1.47 (1.50–1.47)	37.13–1.49 (1.52–1.49)	37.11–1.47 (1.50–1.47)	37.11–1.49 (1.52–1.49)
Number of reflections	6612099	3873108	4157075	3042546	3239469	2877548	3094253
Unique reflections	3846	3487	3484	3477	3365	3495	3360
Redundancy	2736.5 (1719.2)	1718.5 (1110.7)	1906.5 (1193.2)	1352.76 (875.0)	1510.79 (962.7)	1231.3 (823.3)	1380.4 (920.9)
Completeness (%)	100	100	100	100	100	100	100
〈*I*/σ(*I*)〉	13.1 (1.0)	11.54 (1.11)	11.92 (1.22)	9.45 (0.83)	10.08 (1.04)	9.89 (0.85)	10.67 (1.36)
CC^*^	0.9988 (0.6806)	0.9982 (0.7503)	0.9985 (0.6715)	0.9974 (0.7218)	0.9977 (0.7199)	0.9972 (0.7414)	0.9979 (0.7737)
CC_1/2_	0.9954 (0.3013)	0.9928 (0.3917)	0.9941 (0.2911)	0.9899 (0.3523)	0.9907 (0.3492)	0.9887 (0.3790)	0.9917 (0.4271)
*R*_split_ (%)	4.97 (25.57)	6.40 (31.17)	6.13 (32.13)	7.16 (32.93)	7.04 (32.09)	7.29 (32.21)	6.82 (29.77)
Wilson *B* factor (Å^2^)	19.21	19.09	17.62	20.53	19.48	18.17	17.60

**Table 2:** Sample consumption in pump–probe experiments

Protein [pulse mode of XFEL]	Method	Sample used per 10000 indexed images
Photoactivable yellow protein [120 Hz] (Pande *et al.*, 2016 ▸; Pandey *et al.*, 2020 ▸)	GDVN	75 mg
Bacteriorhodopsin [120 Hz] (Nogly *et al.*, 2018 ▸), halorhodopsin [50 Hz] (Mous *et al.*, 2022 ▸)	HVE (LCP)	2.0–2.6 mg
Isocyanide hydratase [n/a] (Dasgupta *et al.*, 2019 ▸)	CoMESH	2.5–3.0 mg
LOV domain 1 [100 Hz]	MISP chip	0.20 mg

## References

[bb1] Botha, S., Nass, K., Barends, T. R. M., Kabsch, W., Latz, B., Dworkowski, F., Foucar, L., Panepucci, E., Wang, M., Shoeman, R. L., Schlichting, I. & Doak, R. B. (2015). *Acta Cryst.* D**71**, 387–397. 10.1107/S1399004714026327 25664750

[bb2] Boutet, S., Lomb, L., Williams, G. J., Barends, T. R., Aquila, A., Doak, R. B., Weierstall, U., DePonte, D. P., Steinbrener, J., Shoeman, R. L., Messerschmidt, M., Barty, A., White, T. A., Kassemeyer, S., Kirian, R. A., Seibert, M. M., Montanez, P. A., Kenney, C., Herbst, R., Hart, P., Pines, J., Haller, G., Gruner, S. M., Philipp, H. T., Tate, M. W., Hromalik, M., Koerner, L. J., van Bakel, N., Morse, J., Ghonsalves, W., Arnlund, D., Bogan, M. J., Caleman, C., Fromme, R., Hampton, C. Y., Hunter, M. S., Johansson, L. C., Katona, G., Kupitz, C., Liang, M., Martin, A. V., Nass, K., Redecke, L., Stellato, F., Timneanu, N., Wang, D., Zatsepin, N. A., Schafer, D., Defever, J., Neutze, R., Fromme, P., Spence, J. C., Chapman, H. N. & Schlichting, I. (2012). *Science*, **337**, 362–364.

[bb3] Brändén, G. & Neutze, R. (2021). *Science*, **373**, eaba0954. 10.1126/science.aba0954 34446579

[bb4] Carrillo, M., Beale, J. & Padeste, C. (2022). *Acta Cryst.* A**78**, a279.

[bb5] Carrillo, M., Mason, T., Karpik, A., Martiel, I., Kepa, M., McAuley, K., Beale, J. & Padeste, C. (2023). *IUCrJ*, **10**, 678–693. 10.1107/S2052252523007595 PMC10619457 37727961

[bb6] Chapman, H. N., Fromme, P., Barty, A., White, T. A., Kirian, R. A., Aquila, A., Hunter, M. S., Schulz, J., DePonte, D. P., Weierstall, U., Doak, R. B., Maia, F. R., Martin, A. V., Schlichting, I., Lomb, L., Coppola, N., Shoeman, R. L., Epp, S. W., Hartmann, R., Rolles, D., Rudenko, A., Foucar, L., Kimmel, N., Weidenspointner, G., Holl, P., Liang, M., Barthelmess, M., Caleman, C., Boutet, S., Bogan, M. J., Krzywinski, J., Bostedt, C., Bajt, S., Gumprecht, L., Rudek, B., Erk, B., Schmidt, C., Hömke, A., Reich, C., Pietschner, D., Strüder, L., Hauser, G., Gorke, H., Ullrich, J., Herrmann, S., Schaller, G., Schopper, F., Soltau, H., Kühnel, K. U., Messerschmidt, M., Bozek, J. D., Hau-Riege, S. P., Frank, M., Hampton, C. Y., Sierra, R. G., Starodub, D., Williams, G. J., Hajdu, J., Timneanu, N., Seibert, M. M., Andreasson, J., Rocker, A., Jönsson, O., Svenda, M., Stern, S., Nass, K., Andritschke, R., Schröter, C. D., Krasniqi, F., Bott, M., Schmidt, K. E., Wang, X., Grotjohann, I., Holton, J. M., Barends, T. R., Neutze, R., Marchesini, S., Fromme, R., Schorb, S., Rupp, D., Adolph, M., Gorkhover, T., Andersson, I., Hirsemann, H., Potdevin, G., Graafsma, H., Nilsson, B. & Spence, J. C. (2011). *Nature*, **470**, 73–77.

[bb7] Claesson, E., Wahlgren, W. Y., Takala, H., Pandey, S., Castillon, L., Kuznetsova, V., Henry, L., Panman, M., Carrillo, M., Kübel, J., Nanekar, R., Isaksson, L., Nimmrich, A., Cellini, A., Morozov, D., Maj, M., Kurttila, M., Bosman, R., Nango, E., Tanaka, R., Tanaka, T., Fangjia, L., Iwata, S., Owada, S., Moffat, K., Groenhof, G., Stojković, E. A., Ihalainen, J. A., Schmidt, M. & Westenhoff, S. (2020). *eLife*, **9**, e53514.

[bb8] Dasgupta, M., Budday, D., de Oliveira, S., Madzelan, P., Marchany-Rivera, D., Seravalli, J., Hayes, B., Sierra, R. G., Boutet, S., Hunter, M. S., Alonso-Mori, R., Batyuk, A., Wierman, J., Lyubimov, A., Brewster, A. S., Sauter, N. K., Applegate, G. A., Tiwari, V. K., Berkowitz, D. B., Thompson, M. C., Cohen, A. E., Fraser, J. S., Wall, M. E., van den Bedem, H. & Wilson, M. A. (2019). *Proc. Natl Acad. Sci. USA*, **116**, 25634–25640. 10.1073/pnas.1901864116 PMC6926069 31801874

[bb9] DeLano, W. (2002). *PyMOL*. https://www.pymol.org.

[bb10] DePonte, D., Doak, R., Hunter, M., Liu, Z., Weierstall, U. & Spence, J. (2009). *Micron*, **40**, 507–509. 10.1016/j.micron.2008.12.009 19246201

[bb11] Doak, R. B., Nass Kovacs, G., Gorel, A., Foucar, L., Barends, T. R. M., Grünbein, M. L., Hilpert, M., Kloos, M., Roome, C. M., Shoeman, R. L., Stricker, M., Tono, K., You, D., Ueda, K., Sherrell, D. A., Owen, R. L. & Schlichting, I. (2018). *Acta Cryst.* D**74**, 1000–1007. 10.1107/S2059798318011634 PMC6173051 30289410

[bb12] Ebrahim, A., Appleby, M. V., Axford, D., Beale, J., Moreno-Chicano, T., Sherrell, D. A., Strange, R. W., Hough, M. A. & Owen, R. L. (2019). *Acta Cryst.* D**75**, 151–159. 10.1107/S2059798318010240 PMC6400251 30821704

[bb13] Fedorov, R., Schlichting, I., Hartmann, E., Domratcheva, T., Fuhrmann, M. & Hegemann, P. (2003). *Biophys. J.* **84**, 2474–2482. 10.1016/S0006-3495(03)75052-8 PMC1302813 12668455

[bb14] Fromme, R., Ishchenko, A., Metz, M., Chowdhury, S. R., Basu, S., Boutet, S., Fromme, P., White, T. A., Barty, A., Spence, J. C. H., Weierstall, U., Liu, W. & Cherezov, V. (2015). *IUCrJ*, **2**, 545–551. 10.1107/S2052252515013160 PMC4547822 26306196

[bb15] Gevorkov, Y., Yefanov, O., Barty, A., White, T. A., Mariani, V., Brehm, W., Tolstikova, A., Grigat, R.-R. & Chapman, H. N. (2019). *Acta Cryst.* A**75**, 694–704. 10.1107/S2053273319010593 PMC6718201 31475914

[bb16] Gisriel, C., Coe, J., Letrun, R., Yefanov, O. M., Luna-Chavez, C., Stander, N. E., Lisova, S., Mariani, V., Kuhn, M., Aplin, S., Grant, T. D., Dörner, K., Sato, T., Echelmeier, A., Cruz Villarreal, J., Hunter, M. S., Wiedorn, M. O., Knoska, J., Mazalova, V., Roy-Chowdhury, S., Yang, J. H., Jones, A., Bean, R., Bielecki, J., Kim, Y., Mills, G., Weinhausen, B., Meza, J. D., Al-Qudami, N., Bajt, S., Brehm, G., Botha, S., Boukhelef, D., Brockhauser, S., Bruce, B. D., Coleman, M. A., Danilevski, C., Discianno, E., Dobson, Z., Fangohr, H., Martin-Garcia, J. M., Gevorkov, Y., Hauf, S., Hosseinizadeh, A., Januschek, F., Ketawala, G. K., Kupitz, C., Maia, L., Manetti, M., Messerschmidt, M., Michelat, T., Mondal, J., Ourmazd, A., Previtali, G., Sarrou, I., Schön, S., Schwander, P., Shelby, M. L., Silenzi, A., Sztuk-Dambietz, J., Szuba, J., Turcato, M., White, T. A., Wrona, K., Xu, C., Abdellatif, M. H., Zook, J. D., Spence, J. C. H., Chapman, H. N., Barty, A., Kirian, R. A., Frank, M., Ros, A., Schmidt, M., Fromme, R., Mancuso, A. P., Fromme, P. & Zatsepin, N. A. (2019). *Nat. Commun.* **10**, 5021.

[bb17] Gotthard, G., Mous, S., Weinert, T., Maia, R. N. A., James, D., Dworkowski, F., Gashi, D., Furrer, A., Ozerov, D., Panepucci, E., Wang, M., Schertler, G. F. X., Heberle, J., Standfuss, J. & Nogly, P. (2023). *bioRxiv*, 2023.11.06.565770.

[bb18] Grünbein, M. L., Bielecki, J., Gorel, A., Stricker, M., Bean, R., Cammarata, M., Dörner, K., Fröhlich, L., Hartmann, E., Hauf, S., Hilpert, M., Kim, Y., Kloos, M., Letrun, R., Messerschmidt, M., Mills, G., Nass Kovacs, G., Ramilli, M., Roome, C. M., Sato, T., Scholz, M., Sliwa, M., Sztuk-Dambietz, J., Weik, M., Weinhausen, B., Al-Qudami, N., Boukhelef, D., Brockhauser, S., Ehsan, W., Emons, M., Esenov, S., Fangohr, H., Kaukher, A., Kluyver, T., Lederer, M., Maia, L., Manetti, M., Michelat, T., Münnich, A., Pallas, F., Palmer, G., Previtali, G., Raab, N., Silenzi, A., Szuba, J., Venkatesan, S., Wrona, K., Zhu, J., Doak, R. B., Shoeman, R. L., Foucar, L., Colletier, J. P., Mancuso, A. P., Barends, T. R. M., Stan, C. A. & Schlichting, I. (2018). *Nat. Commun.* **9**, 3487.

[bb19] Grünbein, M. L., Stricker, M., Nass Kovacs, G., Kloos, M., Doak, R. B., Shoeman, R. L., Reinstein, J., Lecler, S., Haacke, S. & Schlichting, I. (2020). *Nat. Methods*, **17**, 681–684. 10.1038/s41592-020-0847-3 32451477

[bb20] Holzer, W., Penzkofer, A., Fuhrmann, M. & Hegemann, P. (2002). *Photochem. Photobiol.* **75**, 479–487. 10.1562/0031-8655(2002)075<0479:scofmb>2.0.co;2 12017473

[bb21] Hosaka, T., Nomura, T., Kubo, M., Nakane, T., Fangjia, L., Sekine, S. I., Ito, T., Murayama, K., Ihara, K., Ehara, H., Kashiwagi, K., Katsura, K., Akasaka, R., Hisano, T., Tanaka, T., Tanaka, R., Arima, T., Yamashita, A., Sugahara, M., Naitow, H., Matsuura, Y., Yoshizawa, S., Tono, K., Owada, S., Nureki, O., Kimura-Someya, T., Iwata, S., Nango, E. & Shirouzu, M. (2022). *Proc. Natl Acad. Sci. USA*, pp. 119. 10.1073/pnas.2117433119 PMC8892520 35197289

[bb22] Hunter, M. S., Segelke, B., Messerschmidt, M., Williams, G. J., Zatsepin, N. A., Barty, A., Benner, W. H., Carlson, D. B., Coleman, M., Graf, A., Hau-Riege, S. P., Pardini, T., Seibert, M. M., Evans, J., Boutet, S. & Frank, M. (2014). *Sci. Rep.* **4**, 6026. 10.1038/srep06026 PMC4129423 25113598

[bb23] James, D., Weinert, T., Skopintsev, P., Furrer, A., Gashi, D., Tanaka, T., Nango, E., Nogly, P. & Standfuss, J. (2019). *J. Vis. Exp.* pp. e59087. 10.3791/59087 30882786

[bb24] Kaminski, J. W., Vera, L., Stegmann, D., Vering, J., Eris, D., Smith, K. M. L., Huang, C.-Y., Meier, N., Steuber, J., Wang, M., Fritz, G., Wojdyla, J. A. & Sharpe, M. E. (2022). *Acta Cryst.* D**78**, 328–336. 10.1107/S2059798322000705 PMC8900825 35234147

[bb25] Kottke, T., Heberle, J., Hehn, D., Dick, B. & Hegemann, P. (2003). *Biophys. J.* **84**, 1192–1201. 10.1016/S0006-3495(03)74933-9 PMC1302694 12547798

[bb26] Li, H., Nakajima, Y., Nomura, T., Sugahara, M., Yonekura, S., Chan, S. K., Nakane, T., Yamane, T., Umena, Y., Suzuki, M., Masuda, T., Motomura, T., Naitow, H., Matsuura, Y., Kimura, T., Tono, K., Owada, S., Joti, Y., Tanaka, R., Nango, E., Akita, F., Kubo, M., Iwata, S., Shen, J.-R. & Suga, M. (2021). *IUCrJ*, **8**, 431–443. 10.1107/S2052252521002177 PMC8086164 33953929

[bb27] Liebschner, D., Afonine, P. V., Baker, M. L., Bunkóczi, G., Chen, V. B., Croll, T. I., Hintze, B., Hung, L.-W., Jain, S., McCoy, A. J., Moriarty, N. W., Oeffner, R. D., Poon, B. K., Prisant, M. G., Read, R. J., Richardson, J. S., Richardson, D. C., Sammito, M. D., Sobolev, O. V., Stockwell, D. H., Terwilliger, T. C., Urzhumtsev, A. G., Videau, L. L., Williams, C. J. & Adams, P. D. (2019). *Acta Cryst.* D**75**, 861–877. 10.1107/S2059798319011471 PMC6778852 31588918

[bb28] Liu, X., Liu, P., Li, H., Xu, Z., Jia, L., Xia, Y., Yu, M., Tang, W., Zhu, X., Chen, C., Zhang, Y., Nango, E., Tanaka, R., Luo, F., Kato, K., Nakajima, Y., Kishi, S., Yu, H., Matsubara, N., Owada, S., Tono, K., Iwata, S., Yu, L. J., Shen, J. R. & Wang, J. (2022). *Nat. Chem.* **14**, 1054–1060. 10.1038/s41557-022-00992-3 35851837

[bb29] Madden, J. T., Toth, S. J., Dettmar, C. M., Newman, J. A., Oglesbee, R. A., Hedderich, H. G., Everly, R. M., Becker, M., Ronau, J. A., Buchanan, S. K., Cherezov, V., Morrow, M. E., Xu, S., Ferguson, D., Makarov, O., Das, C., Fischetti, R. & Simpson, G. J. (2013). *J. Synchrotron Rad.* **20**, 531–540. 10.1107/S0909049513007942 PMC3682636 23765294

[bb30] Maestre-Reyna, M., Yang, C. H., Nango, E., Huang, W. C., Ngurah Putu, E. P. G., Wu, W. J., Wang, P. H., Franz-Badur, S., Saft, M., Emmerich, H. J., Wu, H. Y., Lee, C. C., Huang, K. F., Chang, Y. K., Liao, J. H., Weng, J. H., Gad, W., Chang, C. W., Pang, A. H., Sugahara, M., Owada, S., Hosokawa, Y., Joti, Y., Yamashita, A., Tanaka, R., Tanaka, T., Luo, F., Tono, K., Hsu, K. C., Kiontke, S., Schapiro, I., Spadaccini, R., Royant, A., Yamamoto, J., Iwata, S., Essen, L. O., Bessho, Y. & Tsai, M. D. (2022). *Nat. Chem.* **14**, 677–685. 10.1038/s41557-022-00922-3 35393554

[bb31] Mehrabi, P., Müller-Werkmeister, H. M., Leimkohl, J.-P., Schikora, H., Ninkovic, J., Krivokuca, S., Andriček, L., Epp, S. W., Sherrell, D., Owen, R. L., Pearson, A. R., Tellkamp, F., Schulz, E. C. & Miller, R. J. D. (2020). *J. Synchrotron Rad.* **27**, 360–370. 10.1107/S1600577520000685 PMC7064102 32153274

[bb32] Mehrabi, P., Schulz, E. C., Agthe, M., Horrell, S., Bourenkov, G., von Stetten, D., Leimkohl, J. P., Schikora, H., Schneider, T. R., Pearson, A. R., Tellkamp, F. & Miller, R. J. D. (2019). *Nat. Methods*, **16**, 979–982. 10.1038/s41592-019-0553-1 31527838

[bb33] Mehrabi, P., Schulz, E. C., Dsouza, R., Müller-Werkmeister, H. M., Tellkamp, F., Miller, R. J. D. & Pai, E. F. (2019). *Science*, **365**, 1167–1170. 10.1126/science.aaw9904 31515393

[bb34] Mous, S., Gotthard, G., Ehrenberg, D., Sen, S., Weinert, T., Johnson, P. J., James, D., Nass, K., Furrer, A., Kekilli, D., Ma, P., Brünle, S., Casadei, C. M., Martiel, I., Dworkowski, F., Gashi, D., Skopintsev, P., Wranik, M., Knopp, G., Panepucci, E., Panneels, V., Cirelli, C., Ozerov, D., Schertler, G. F. X., Wang, M., Milne, C., Standfuss, J., Schapiro, I., Heberle, J. & Nogly, P. (2022). *Science*, **375**, 845–851. 10.1126/science.abj6663 35113649

[bb35] Mueller, C., Marx, A., Epp, S. W., Zhong, Y., Kuo, A., Balo, A. R., Soman, J., Schotte, F., Lemke, H. T., Owen, R. L., Pai, E. F., Pearson, A. R., Olson, J. S., Anfinrud, P. A., Ernst, O. P. & Dwayne Miller, R. J. (2015). *Struct. Dyn.* **2**, 054302. 10.1063/1.4928706 PMC4711646 26798825

[bb36] Nam, K. H., Kim, J. & Cho, Y. (2021). *Sci. Rep.* **11**, 13115. 10.1038/s41598-021-92687-x PMC8222285 34162965

[bb37] Nango, E., Royant, A., Kubo, M., Nakane, T., Wickstrand, C., Kimura, T., Tanaka, T., Tono, K., Song, C., Tanaka, R., Arima, T., Yamashita, A., Kobayashi, J., Hosaka, T., Mizohata, E., Nogly, P., Sugahara, M., Nam, D., Nomura, T., Shimamura, T., Im, D., Fujiwara, T., Yamanaka, Y., Jeon, B., Nishizawa, T., Oda, K., Fukuda, M., Andersson, R., Båth, P., Dods, R., Davidsson, J., Matsuoka, S., Kawatake, S., Murata, M., Nureki, O., Owada, S., Kameshima, T., Hatsui, T., Joti, Y., Schertler, G. F. X., Yabashi, M., Bondar, A.-N., Standfuss, J., Neutze, R. & Iwata, S. (2016). *Science*, **354**, 1552–1557. 10.1126/science.aah3497 28008064

[bb38] Nass Kovacs, G., Colletier, J. P., Grünbein, M. L., Yang, Y., Stensitzki, T., Batyuk, A., Carbajo, S., Doak, R. B., Ehrenberg, D., Foucar, L., Gasper, R., Gorel, A., Hilpert, M., Kloos, M., Koglin, J. E., Reinstein, J., Roome, C. M., Schlesinger, R., Seaberg, M., Shoeman, R. L., Stricker, M., Boutet, S., Haacke, S., Heberle, J., Heyne, K., Domratcheva, T., Barends, T. R. M. & Schlichting, I. (2019). *Nat. Commun.* **10**, 3177. 10.1038/s41467-019-10758-0 PMC6639342 31320619

[bb39] Neutze, R., Wouts, R., van der Spoel, D., Weckert, E. & Hajdu, J. (2000). *Nature*, **406**, 752–757. 10.1038/35021099 10963603

[bb40] Nogly, P., James, D., Wang, D., White, T. A., Zatsepin, N., Shilova, A., Nelson, G., Liu, H., Johansson, L., Heymann, M., Jaeger, K., Metz, M., Wickstrand, C., Wu, W., Båth, P., Berntsen, P., Oberthuer, D., Panneels, V., Cherezov, V., Chapman, H., Schertler, G., Neutze, R., Spence, J., Moraes, I., Burghammer, M., Standfuss, J. & Weierstall, U. (2015). *IUCrJ*, **2**, 168–176. 10.1107/S2052252514026487 PMC4392771 25866654

[bb41] Nogly, P., Panneels, V., Nelson, G., Gati, C., Kimura, T., Milne, C., Milathianaki, D., Kubo, M., Wu, W., Conrad, C., Coe, J., Bean, R., Zhao, Y., Båth, P., Dods, R., Harimoorthy, R., Beyerlein, K. R., Rheinberger, J., James, D., DePonte, D., Li, C., Sala, L., Williams, G. J., Hunter, M. S., Koglin, J. E., Berntsen, P., Nango, E., Iwata, S., Chapman, H. N., Fromme, P., Frank, M., Abela, R., Boutet, S., Barty, A., White, T. A., Weierstall, U., Spence, J., Neutze, R., Schertler, G. & Standfuss, J. (2016). *Nat. Commun.* **7**, 12314. 10.1038/ncomms12314 PMC4996941 27545823

[bb42] Nogly, P., Weinert, T., James, D., Carbajo, S., Ozerov, D., Furrer, A., Gashi, D., Borin, V., Skopintsev, P., Jaeger, K., Nass, K., Bath, P., Bosman, R., Koglin, J., Seaberg, M., Lane, T., Kekilli, D., Bruenle, S., Tanaka, T., Wu, W., Milne, C., White, T. A., Barty, A., Weierstall, U., Panneels, V., Nango, E., Iwata, S., Hunter, M., Schapiro, I., Schertler, G. F. X., Neutze, R. & Standfuss, J. (2018). *Science*, pp. 361 eaat0094. 10.1126/science.aat0094 29903883

[bb43] Oghbaey, S., Sarracini, A., Ginn, H. M., Pare-Labrosse, O., Kuo, A., Marx, A., Epp, S. W., Sherrell, D. A., Eger, B. T., Zhong, Y., Loch, R., Mariani, V., Alonso-Mori, R., Nelson, S., Lemke, H. T., Owen, R. L., Pearson, A. R., Stuart, D. I., Ernst, O. P., Mueller-Werkmeister, H. M. & Miller, R. J. D. (2016). *Acta Cryst.* D**72**, 944–955. 10.1107/S2059798316010834 PMC5937680 27487825

[bb44] Olmos, J. L. Jr, Pandey, S., Martin-Garcia, J. M., Calvey, G., Katz, A., Knoska, J., Kupitz, C., Hunter, M. S., Liang, M., Oberthuer, D., Yefanov, O., Wiedorn, M., Heyman, M., Holl, M., Pande, K., Barty, A., Miller, M. D., Stern, S., Roy-Chowdhury, S., Coe, J., Nagaratnam, N., Zook, J., Verburgt, J., Norwood, T., Poudyal, I., Xu, D., Koglin, J., Seaberg, M. H., Zhao, Y., Bajt, S., Grant, T., Mariani, V., Nelson, G., Subramanian, G., Bae, E., Fromme, R., Fung, R., Schwander, P., Frank, M., White, T. A., Weierstall, U., Zatsepin, N., Spence, J., Fromme, P., Chapman, H. N., Pollack, L., Tremblay, L., Ourmazd, A., Phillips, G. N. Jr & Schmidt, M. (2018). *BMC Biol.* **16**, 59. 10.1186/s12915-018-0524-5 PMC5977757 29848358

[bb45] Pande, K., Hutchison, C., Groenhof, G., Aquila, A., Robinson, J., Tenboer, J., Basu, S., Boutet, S., DePonte, D. P., Liang, M., White, T. A., Zatsepin, N., Yefanov, O., Morozov, D., Oberthuer, D., Gati, C., Subramanian, G., James, D., Zhao, Y., Koralek, J., Brayshaw, J., Kupitz, C., Conrad, C., Roy-Chowdhury, S., Coe, J. D., Metz, M., Xavier, P. L., Grant, T. D., Koglin, J., Ketawala, G., Fromme, R., Šrajer, V., Henning, R., Spence, J. C., Ourmazd, A., Schwander, P., Weierstall, U., Frank, M., Fromme, P., Barty, A., Chapman, H. N., Moffat, K., van Thor, J. J. & Schmidt, M. (2016). *Science*, **352**, 725–729. 10.1126/science.aad5081 PMC5291079 27151871

[bb46] Pandey, S., Bean, R., Sato, T., Poudyal, I., Bielecki, J., Cruz Villarreal, J., Yefanov, O., Mariani, V., White, T. A., Kupitz, C., Hunter, M., Abdellatif, M. H., Bajt, S., Bondar, V., Echelmeier, A., Doppler, D., Emons, M., Frank, M., Fromme, R., Gevorkov, Y., Giovanetti, G., Jiang, M., Kim, D., Kim, Y., Kirkwood, H., Klimovskaia, A., Knoska, J., Koua, F. H. M., Letrun, R., Lisova, S., Maia, L., Mazalova, V., Meza, D., Michelat, T., Ourmazd, A., Palmer, G., Ramilli, M., Schubert, R., Schwander, P., Silenzi, A., Sztuk-Dambietz, J., Tolstikova, A., Chapman, H. N., Ros, A., Barty, A., Fromme, P., Mancuso, A. P. & Schmidt, M. (2020). *Nat. Methods*, **17**, 73–78. 10.1038/s41592-019-0628-z PMC9113060 31740816

[bb47] Park, S.-Y., Choi, H., Eo, C., Cho, Y. & Nam, K. (2020). *Crystals*, **10**, 803.

[bb48] Pompidor, G., Dworkowski, F. S. N., Thominet, V., Schulze-Briese, C. & Fuchs, M. R. (2013). *J. Synchrotron Rad.* **20**, 765–776. 10.1107/S0909049513016063 PMC3747950 23955041

[bb49] Schulz, E. C., Yorke, B. A., Pearson, A. R. & Mehrabi, P. (2022). *Acta Cryst.* D**78**, 14–29. 10.1107/S2059798321011621 PMC8725164 34981758

[bb50] Sherrell, D. A., Foster, A. J., Hudson, L., Nutter, B., O’Hea, J., Nelson, S., Paré-Labrosse, O., Oghbaey, S., Miller, R. J. D. & Owen, R. L. (2015). *J. Synchrotron Rad.* **22**, 1372–1378. 10.1107/S1600577515016938 PMC4629865 26524301

[bb51] Sherrell, D. A., Lavens, A., Wilamowski, M., Kim, Y., Chard, R., Lazarski, K., Rosenbaum, G., Vescovi, R., Johnson, J. L., Akins, C., Chang, C., Michalska, K., Babnigg, G., Foster, I. & Joachimiak, A. (2022). *J. Synchrotron Rad.* **29**, 1141–1151. 10.1107/S1600577522007895 PMC9455217 36073872

[bb52] Sierra, R. G., Laksmono, H., Kern, J., Tran, R., Hattne, J., Alonso-Mori, R., Lassalle-Kaiser, B., Glöckner, C., Hellmich, J., Schafer, D. W., Echols, N., Gildea, R. J., Grosse-Kunstleve, R. W., Sellberg, J., McQueen, T. A., Fry, A. R., Messerschmidt, M. M., Miahnahri, A., Seibert, M. M., Hampton, C. Y., Starodub, D., Loh, N. D., Sokaras, D., Weng, T.-C., Zwart, P. H., Glatzel, P., Milathianaki, D., White, W. E., Adams, P. D., Williams, G. J., Boutet, S., Zouni, A., Messinger, J., Sauter, N. K., Bergmann, U., Yano, J., Yachandra, V. K. & Bogan, M. J. (2012). *Acta Cryst.* D**68**, 1584–1587.

[bb53] Skopintsev, P., Ehrenberg, D., Weinert, T., James, D., Kar, R. K., Johnson, P. J. M., Ozerov, D., Furrer, A., Martiel, I., Dworkowski, F., Nass, K., Knopp, G., Cirelli, C., Arrell, C., Gashi, D., Mous, S., Wranik, M., Gruhl, T., Kekilli, D., Brünle, S., Deupi, X., Schertler, G. F. X., Benoit, R. M., Panneels, V., Nogly, P., Schapiro, I., Milne, C., Heberle, J. & Standfuss, J. (2020). *Nature*, **583**, 314–318. 10.1038/s41586-020-2307-8 32499654

[bb54] Smolentsev, G., Guda, A., Zhang, X., Haldrup, K., Andreiadis, E., Chavarot-Kerlidou, M., Canton, S. E., Nachtegaal, M., Artero, V. & Sundstrom, V. (2013). *J. Phys. Chem. C*, **117**, 17367–17375. 10.1021/jp4010554 PMC3892145 24443663

[bb55] Studier, F. (2005). *Protein Expr. Purif.* **41**, 207–234. 10.1016/j.pep.2005.01.016 15915565

[bb56] Suga, M., Akita, F., Sugahara, M., Kubo, M., Nakajima, Y., Nakane, T., Yamashita, K., Umena, Y., Nakabayashi, M., Yamane, T., Nakano, T., Suzuki, M., Masuda, T., Inoue, S., Kimura, T., Nomura, T., Yonekura, S., Yu, L. J., Sakamoto, T., Motomura, T., Chen, J. H., Kato, Y., Noguchi, T., Tono, K., Joti, Y., Kameshima, T., Hatsui, T., Nango, E., Tanaka, R., Naitow, H., Matsuura, Y., Yamashita, A., Yamamoto, M., Nureki, O., Yabashi, M., Ishikawa, T., Iwata, S. & Shen, J. R. (2017). *Nature*, **543**, 131–135. 10.1038/nature21400 28219079

[bb57] Weierstall, U. (2014). *Phil. Trans. R. Soc. B*, **369**, 20130337. 10.1098/rstb.2013.0337 PMC4052872 24914163

[bb58] Weierstall, U., James, D., Wang, C., White, T. A., Wang, D., Liu, W., Spence, J. C. H., Bruce Doak, R., Nelson, G., Fromme, P., Fromme, R., Grotjohann, I., Kupitz, C., Zatsepin, N. A., Liu, H., Basu, S., Wacker, D., Won Han, G., Katritch, V., Boutet, S., Messerschmidt, M., Williams, G. J., Koglin, J., Marvin Seibert, M., Klinker, M., Gati, C., Shoeman, R. L., Barty, A., Chapman, H. N., Kirian, R. A., Beyerlein, K. R., Stevens, R. C., Li, D., Shah, S. T. A., Howe, N., Caffrey, M. & Cherezov, V. (2014). *Nat. Commun.* **5**, 3309. 10.1038/ncomms4309 PMC4061911 24525480

[bb59] White, T. A. (2019). *Acta Cryst.* D**75**, 219–233. 10.1107/S205979831801238X PMC6400257 30821710

[bb60] Wiedorn, M. O., Oberthür, D., Bean, R., Schubert, R., Werner, N., Abbey, B., Aepfelbacher, M., Adriano, L., Allahgholi, A., Al-Qudami, N., Andreasson, J., Aplin, S., Awel, S., Ayyer, K., Bajt, S., Barák, I., Bari, S., Bielecki, J., Botha, S., Boukhelef, D., Brehm, W., Brockhauser, S., Cheviakov, I., Coleman, M. A., Cruz-Mazo, F., Danilevski, C., Darmanin, C., Doak, R. B., Domaracky, M., Dörner, K., Du, Y., Fangohr, H., Fleckenstein, H., Frank, M., Fromme, P., Gañán-Calvo, A. M., Gevorkov, Y., Giewekemeyer, K., Ginn, H. M., Graafsma, H., Graceffa, R., Greiffenberg, D., Gumprecht, L., Göttlicher, P., Hajdu, J., Hauf, S., Heymann, M., Holmes, S., Horke, D. A., Hunter, M. S., Imlau, S., Kaukher, A., Kim, Y., Klyuev, A., Knoška, J., Kobe, B., Kuhn, M., Kupitz, C., Küpper, J., Lahey-Rudolph, J. M., Laurus, T., Le Cong, K., Letrun, R., Xavier, P. L., Maia, L., Maia, F., Mariani, V., Messerschmidt, M., Metz, M., Mezza, D., Michelat, T., Mills, G., Monteiro, D. C. F., Morgan, A., Mühlig, K., Munke, A., Münnich, A., Nette, J., Nugent, K. A., Nuguid, T., Orville, A. M., Pandey, S., Pena, G., Villanueva-Perez, P., Poehlsen, J., Previtali, G., Redecke, L., Riekehr, W. M., Rohde, H., Round, A., Safenreiter, T., Sarrou, I., Sato, T., Schmidt, M., Schmitt, B., Schönherr, R., Schulz, J., Sellberg, J. A., Seibert, M. M., Seuring, C., Shelby, M. L., Shoeman, R. L., Sikorski, M., Silenzi, A., Stan, C. A., Shi, X., Stern, S., Sztuk-Dambietz, J., Szuba, J., Tolstikova, A., Trebbin, M., Trunk, U., Vagovic, P., Ve, T., Weinhausen, B., White, T. A., Wrona, K., Xu, C., Yefanov, O., Zatsepin, N., Zhang, J., Perbandt, M., Mancuso, A. P., Betzel, C., Chapman, H. & Barty, A. (2018). *Nat. Commun.* **9**, 4025.

[bb61] Winn, M. D., Ballard, C. C., Cowtan, K. D., Dodson, E. J., Emsley, P., Evans, P. R., Keegan, R. M., Krissinel, E. B., Leslie, A. G. W., McCoy, A., McNicholas, S. J., Murshudov, G. N., Pannu, N. S., Potterton, E. A., Powell, H. R., Read, R. J., Vagin, A. & Wilson, K. S. (2011). *Acta Cryst.* D**67**, 235–242. 10.1107/S0907444910045749 PMC3069738 21460441

[bb62] Wolff, A. M., Nango, E., Young, I. D., Brewster, A. S., Kubo, M., Nomura, T., Sugahara, M., Owada, S., Barad, B. A., Ito, K., Bhowmick, A., Carbajo, S., Hino, T., Holton, J. M., Im, D., O’Riordan, L. J., Tanaka, T., Tanaka, R., Sierra, R. G., Yumoto, F., Tono, K., Iwata, S., Sauter, N. K., Fraser, J. S. & Thompson, M. C. (2023). *Nat. Chem.* **15**, 1549–1558. 10.1038/s41557-023-01329-4 PMC10624634 37723259

[bb63] Wranik, M., Weinert, T., Slavov, C., Masini, T., Furrer, A., Gaillard, N., Gioia, D., Ferrarotti, M., James, D., Glover, H., Carrillo, M., Kekilli, D., Stipp, R., Skopintsev, P., Brünle, S., Mühlethaler, T., Beale, J., Gashi, D., Nass, K., Ozerov, D., Johnson, P. J. M., Cirelli, C., Bacellar, C., Braun, M., Wang, M., Dworkowski, F., Milne, C., Cavalli, A., Wachtveitl, J., Steinmetz, M. O. & Standfuss, J. (2023). *Nat. Commun.* **14**, 903. 10.1038/s41467-023-36481-5 PMC9936131 36807348

[bb64] Zarrine-Afsar, A., Barends, T. R. M., Müller, C., Fuchs, M. R., Lomb, L., Schlichting, I. & Miller, R. J. D. (2012). *Acta Cryst.* D**68**, 321–323. 10.1107/S0907444911055296 22349234

